# HER2-targeted PET-imaging with [68Ga]Ga-ABY-025 to predict targeted-therapy response in HER2-expressing metastatic breast cancer: study protocol of a multicenter, prospective, open-label trial

**DOI:** 10.3389/fonc.2026.1812711

**Published:** 2026-04-10

**Authors:** Renske Altena, Oscar P. B. Wiklander, Nour Susaeg Romdhani, Anna Kistner, Marie Klintman, Ida Skarping, Henrik Lindman, Veronica Sanchez Rodriguez, Oskar Frisell, Peter Lindgren, Klas Bratteby, Emma Jussing, Joachim Feldwisch, Fredrik Y. Frejd, Helena Silva Cascales, Jonathan Siikanen, Cecilia Hindorf, Thuy A. Tran

**Affiliations:** 1 Department of Oncology-Pathology, Karolinska Institutet, Stockholm, Sweden; 2 Department of Laboratory Medicine, Karolinska Institutet, Stockholm, Sweden; 3 Breast Center, Karolinska Comprehensive Cancer Center, Karolinska University Hospital, Stockholm, Sweden; 4 Department of Molecular Medicine and Surgery, Karolinska Institutet, Stockholm, Sweden; 5 Theranostics Trial Center, Department of Nuclear Medicine and Medical Physics, Karolinska University Hospital, Stockholm, Sweden; 6 Division of Oncology, Department of Clinical Sciences, Lund University, and Skåne University Hospital, Lund, Sweden; 7 Department of Clinical Physiology and Nuclear Medicine, Skane University Hospital, Lund, Sweden; 8 Department of Oncology, Uppsala University hospital, Uppsala, Sweden; 9 Division of Radiology and Nuclear Medicine, Department of Surgical Sciences, Uppsala University, Uppsala, Sweden; 10 The Swedish Institute for Health Economics, Stockholm, Sweden; 11 Department of Learning, Informatics, Management and Ethics, Karolinska Institutet, Solna, Sweden; 12 Affibody AB, Stockholm, Sweden; 13 Department of Immunology, Genetics and Pathology, Uppsala University, Uppsala, Sweden

**Keywords:** [68Ga]Ga-ABY-025, HER2-targeted PET, metastatic HER2-low breast cancer, molecular imaging, predictive biomarker

## Abstract

**Background:**

Trastuzumab deruxtecan (T-DXd) is effective in HER2-expressing metastatic breast cancer (mBC), yet inter-patient benefit varies. The current reference biomarker—HER2 immunohistochemistry (IHC) on tumor biopsies—is insufficient as a sole predictor of response. HER2-targeted PET/CT offers non-invasive, whole-body, real-time assessment of target expression. We hypothesize that HER2-targeted PET with [68Ga]Ga-ABY-025 improves prediction of T-DXd outcomes and supports individualized treatment planning.

**Methods:**

HER2-Ex PET is a multicenter, phase II open-label diagnostic trial enrolling patients with HER2-low mBC who are candidates for T-DXd under current approvals (EU CT 2024-512721-89-00; NCT06830382). All participants undergo baseline HER2-PET with [68Ga]Ga-ABY-025 and a tumor biopsy. Patients with biopsy-confirmed HER2 expression (IHC 1–2+; Cohort 1) receive T-DXd and repeat HER2-PET after 3–4 cycles; others receive physician’s-choice systemic therapy (Cohort 2). The primary endpoint is the association between baseline HER2-PET signal—defined as the mean SUVmax across the five most avid lesions—and objective response per RECIST v1.1 after 3–4 cycles of T-DXd. A total sample size of 70 provides 80% power (α=0.05), allowing for attrition and technical failures. Secondary endpoints include health-economic outcomes and translational analyses of tumor biology and heterogeneity. The study is open at Karolinska University Hospital (Stockholm, Sweden), with Uppsala and Skåne University Hospitals planned for activation in Q1 2026.

**Discussion:**

Demonstrating a robust correlation between HER2-PET signal and early radiologic response would validate imaging-based patient selection for T-DXd, facilitate adaptive treatment decisions, and enhance biological understanding of intra- and inter-patient HER2 heterogeneity. Key considerations include standardization of imaging protocols across sites, potential temporal discordance between biopsy and imaging, and the non-randomized design. Positive results would justify incorporation of HER2-targeted PET into clinical pathways and inform the design of subsequent randomized trials testing PET-guided T-DXd strategies.

**Clinical Trial Registration:**

https://clinicaltrials.gov/study/NCT06830382, identifier NCT06830382.

## Introduction

Human Epidermal Growth Factor Receptor 2 (HER2)-positive breast cancer, defined as an immunohistochemical (IHC) score of 3+ or 2+ with confirmed HER2 gene amplification, occurs in approximately 15% of patients. For these individuals, HER2-targeted therapies remain a cornerstone of treatment. More recently, the recognition of HER2-low breast cancer — characterized by an IHC score of 1+ or 2+ without gene amplification and present in around 50% of patients — has significantly altered clinical perspectives on HER2 as a predictive biomarker (1).

The antibody-drug conjugate (ADC) trastuzumab deruxtecan (T-DXd) has demonstrated improved progression-free survival (PFS) and overall survival (OS) in patients with HER2-low metastatic breast cancer (mBC) who have received at least one prior line of chemotherapy in the palliative setting (2). Intriguingly, emerging evidence suggests that even patients with ultra-low or absent HER2 expression in tumor biopsies may benefit from T-DXd (3, 4).

Currently, HER2 status is determined using fresh or archived tumor biopsies. However, spatial heterogeneity and temporal variation in HER2 expression—as well as inconsistencies in pathological assessments—undermine the reliability of single-site biopsy results (5). Moreover, certain metastatic sites, such as bone lesions, present technical challenges for IHC evaluation, and others may be inaccessible for biopsy. These limitations underscore the need for alternative methods to identify patients who may benefit from HER2-targeted therapies.

HER2-specific radiotracers for positron emission tomography (PET) imaging offer a promising non-invasive alternative. While several HER2-targeted tracers have been evaluated (6), primarily in HER2-positive mBC, a pilot study (NCT05619016) in ten patients with HER2-low mBC demonstrated the feasibility of [^68^Ga]Ga-ABY-025 PET imaging (7). All patients showed tracer uptake in metastatic lesions, with notable intra- and interindividual heterogeneity. In eight of ten cases, HER2-low status was confirmed by IHC/*in situ* hybridization in the biopsied lesion. Interestingly, two patients had IHC 0: one in a cutaneous lesion with low SUVmax and another in a liver metastasis with high SUVmax but a necrotic core. Additionally, HER2-low mBC has also been visualized using zirconium-89-labeled monoclonal antibody tracers (8).

A few trials have explored HER2-targeted PET/CT as a predictor of response to HER2-targeted therapy in HER2-amplified breast cancer patients. [^89^Zr]Zr-trastuzumab PET/CT uptake has been shown to correlate with outcomes on the HER2-ADC trastuzumab emtansine, with PET-positive patients achieving longer time to treatment failure (TTF) than PET-negative patients (9). Similar findings have been reported with [^64^Cu]Cu-DOTA-trastuzumab, where early metabolic responders had markedly prolonged TTF (10). In addition, [^68^Ga]Ga-ABY-025 PET/CT demonstrated that higher baseline uptake was associated with metabolic response on, particularly in metastatic disease (11). Collectively, these data suggest that HER2-targeted PET/CT may serve as a non-invasive biomarker of treatment response for HER2-positive mBC, although prospective validation of the method as a therapy-selection tool is warranted. In order to facilitate implementation of this diagnostic approach into routine care the cost-effectiveness of using [^68^Ga]Ga-ABY-025 PET imaging compared to current standard of care needs to be established. As of today, to the best of our knowledge, this has not been assessed.

Here we present the trial protocol of the HER2-Ex PET trial (EudraCT 2024-512721-89-00; NCT06830382) a phase II diagnostic imaging study designed to further this research. The primary aim is to evaluate [^68^Ga]Ga-ABY-025 PET/CT as a precision imaging tool to refine patient selection for T-DXd therapy among individuals with HER2-low mBC. The study hypothesis is that PET/CT imaging with the HER2-targeted tracer [^68^Ga]Ga-ABY-025 will improve treatment planning and enhance biological understanding of HER2 expression patterns in mBC, ultimately supporting a more tailored and effective therapeutic approach.

## Methods and analysis

### Design

**Figure 1:**
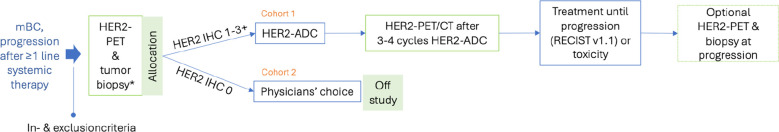
Trial flow chart. mBC, metastatic breast cancer; HER2, Human Epidermal growth factor Receptor 2; PET, positron emission tomography; CT, computed tomography; IHC, Immunohistochemistry; ADC, antibody drug conjugate. *If no archived biopsy <18 months.

To test our hypothesis, we have initiated an investigator-initiated, multicenter, prospective, open-label phase II HER2-targeted PET/CT imaging trial with [^68^Ga]Ga-ABY-025 in patients with HER2-low mBC who are considered candidates for treatment with the HER2-ADC T-DXd. The study design is summarized as SPIRIT schedule of enrolment, interventions, and assessments in **Table 1**. A graphical representation of the study design is presented in **Figure 1**.

**Table 1:** SPIRIT schedule of enrolment, interventions and assessments in the HER2-Ex PET trial.

Study period						
Type of assessment	Enrolment	Allocation		Post-allocation		Close-out
	All patients		Cohort 2 (HER2 0)	Cohort 1	(HER2 expressing)	
TIMEPOINT (weeks)	-(1-2)	0	12	9-12	Disease progression	EoT with T-DXd + min1 y FU
ENROLMENT:						
Eligibility screen	X					
Informed consent	X					
6BGa-ABY-025 tracer injection				X	X	
PET/CT image acquisition	X (3 hours post-injection)			X	X	
Tumor biopsy	X (48 hours after PET)					
INTERVENTIONS:						
Cohort allocation according to HER2 status		X				
ASSESSMENTS:						
CT-thorax-abdomen (RECIST v1.1)				X	X	
Blood sample collection (EVs)				X		
HR QoL (EQ-5D-5L)						
IMP-related AE's			X	X	X	
Vital status						X

HER2, Human Epidermal growth factor Receptor 2; PET/CT, Positron Emission Tomography with Computed Tomography; HR QoL, Health Related Quality of Life; PD, progressive disease; EoT, End of Treatment; T-DXd, trastuzumab deruxtecan; min, minimum; yr, year; FU, follow-up.

The target population in the HER2-Ex PET trial (EU CT number: 2024-512721-89-00; clinicaltrials.gov identifier: NCT06830382) comprises patients with mBC who are eligible for systemic treatment with T-DXd, in accordance with current regulatory approvals for HER2-low mBC. The trial has been approved by the Swedish Ethical Review Authority and Medical Product Agency. The current protocol version used is version 3.0, dated January 22^nd^ 2025. Updates will be communicated with trial sites and published at clinicaltrials.gov.

Participants are recruited from Karolinska University Hospital (Stockholm), Uppsala University Hospital, and Skåne University Hospital (Lund), all located in Sweden. Study-specific procedures—including [^68^Ga]Ga-ABY-025 PET/CT imaging, tumor biopsies, and blood sampling—will be conducted at the respective recruitment sites.

The precursor ABY-025 (Affibody AB, Solna, Sweden), will be radiolabeled with 68-Ga to form the radiotracer [^68^Ga]Ga-ABY-025 at the Departments of Radiopharmacy at Karolinska University Hospital in Solna and Skåne University Hospital in Lund. For Uppsala participants, the tracer will be delivered by courier from Karolinska Radiopharmacy. HER2 IHC will be performed at each site’s certified pathology laboratory, and all samples will undergo central review by the designated study pathologist upon trial completion.

PET imaging will be performed using a harmonized acquisition protocol on GE Discovery MI PET/CT scanners equipped with 5 rings (Uppsala) or 4 detector rings (Lund and Karolinska). All PET images will be collected and centrally reviewed at the end of the study. At the moment of writing (Q3 2025), the HER2-Ex PET trial is opened for recruitment in Stockholm and recruitment is anticipated to start in Uppsala and Lund in Q1 2026.

### Selection and treatment of subjects

Patients with metastatic or unresectable breast cancer are eligible for inclusion in the HER2-Ex PET trial, based on the inclusion and exclusion criteria outlined in **Table 2**. Briefly, participants must be medically fit and willing to receive T-DXd treatment, undergo a tumor biopsy, and participate in PET/CT imaging. Patients who have previously been treated with HER2-targeted drugs cannot be included. Tumor lesions must be measurable according to RECIST v1.1 (12), and at least one non-skeletal lesion must be accessible for ultrasound- or CT-guided biopsy. Patients must have no contraindications to PET/CT or T-DXd therapy.

**Table 2:** Inclusion and exclusion criteria in the HER2 Ex-PET trial.

Inclusion criteria	Exclusion criteria
Age ≥ 18 years Diagnosed with mBC Disease progression after ≥ 1 line of chemotherapy in the palliative setting, or with disease relapse within six months after completion of (neo-) adjuvant chemotherapy. Measurable disease according to RECIST v1.1 At least one metastatic lesion accessible for biopsy (if no biopsy not older than 12 months is available) Deemed by treating physician as fit for systemic therapy according to study protocol ECOG performance score 0 or 1 Adequate blood tests for bone marrow, renal and hepatic functions • Able and willing to provide written informed consent	Contra-indications for treatment for trastuzumab deruxtecan and inability to undergo this treatment as per local treatment routines. A previously documented metastatic tumor biopsy that was HER2-positive (IHC 3+ and/or HER2 gene amplification). Contraindications for PET/CT as defined for clinical practice Other manifest malignancies except for basal cell carcinoma of the skin. Inadequate cardiac, renal, bone marrow, or liver function For women in childbearing age: pregnancy or lactation, or inadequate contraception • Patients with increased risk of complications from biopsies, i.e. increased risk of bleeding

Participants are screened and recruited by PI or its local delegates at each participating site. Written informed consent is required prior to randomization and will be obtained by a physician authorized through appropriate delegation.

### Interventional methods

Study-related assessments include imaging, laboratory tests (blood and tumor samples) and clinical outcome assessments including treatment response and toxicities, as summarized in the SPIRIT chart in **Figure 1**. The investigational Medical Product (IMP) in this trial is [^68^Ga]Ga-ABY-025 PET/CT; T-DXd is Auxiliary Medicinal Product (AMP).

#### Imaging assessments

The IMP [^68^Ga]Ga-ABY-025 will be administered two to three times to each participant in Cohort 1 (if biopsy confirms a HER2-expressing tumor) and once to participants in Cohort 2 (HER2 IHC 0 tumors).

For all participants and scans, the IMP is intravenously administrated three hours prior to PET/CT scanning. The first PET/CT scan is a baseline scan, which is scheduled within 21 days before the initiation of systemic oncological therapy. In Cohort 1 a second follow-up PET/CT scan is conducted in conjunction with radiologic response assessment after three to four cycles of T-DXd, per local routines and decided by the treating physician to facilitate the adherence to the trial. The rationale for performing a second HER2-PET/CT is to evaluate dynamics in HER2-avidity in response to treatment efficacy according to radiological response as well as other disease-related outcomes. An optional third PET scan may be performed upon discontinuation of T-DXd due to disease progression or unacceptable toxicity.

The three-hour uptake period of the IMP before the PET/CT imaging is based on prior studies demonstrating optimal image contrast between two to four hours post-injection (7, 11). Conducting therapy evaluation after 3–4 treatment cycles reflect established clinical practice and allows for early detection of radiologic signs of treatment-induced pneumonitis.

A patient will receive an effective dose of 6 mSv from one [^68^Ga]Ga-ABY-025 injection (rc), 0.3 mSv from one attenuation correction CT and 15.5 mSv from one diagnostic CT.

Radiological response evaluation is done according to RECIST v1.1 (12). For the reporting of the trial results, all evaluation CT-investigations will be reviewed centrally by two dedicated radiologists who are not informed about patient characteristics, treatments given and outcomes.

#### Tumor samples

HER2 status in tumor biopsies will be assessed according to standard clinical procedures at each participating site’s pathology laboratory. Upon completion of patient inclusion, digitized biopsy samples from all participants will be submitted for central review by the study pathologist at Karolinska University Hospital. In cases of discrepancy between the local assessment and the central re-evaluation, the result from the central review will be considered definitive.

#### Blood samples for circulating extracellular vesicles

In conjunction with the HER2-PET investigations, for the patients included in Stockholm, bloodsamples will be drawn to analyze for Extracellular Vesicles (EVs). EVs are a heterogeneous group of lipid-bilayer nanovesicles that are secreted by all cells and can be found in all body fluids. These natural nanovesicles range in size from 30-2,000 nm in diameter and can impact both neighboring cells or cells at distance. EVs contain lipids, proteins, and nucleic acid species from the source cell, and have the unique ability to convey these macromolecules via an advanced system of intercellular communication (13, 14). EVs can be seen as a fingerprint of its source cell, reflecting its status and content, and are thus studied as potential biomarkers of various diseases, with particular focus on cancer. Here, tumor-derived EVs will be isolated from blood. The amount of HER2-positive tumor-EVs and the relative HER2-expression on these EVs will be assessed and correlated to the PET/CT findings and patient outcome. Further proteomic and Next-generation sequencing (NGS) analysis on the EVs will also be conducted to gain insight into disease-related molecular aberrations.

#### Questionnaires for health-related quality of life

At baseline, after the first treatment evaluation (for patients in Cohort 1 after 3–4 cycles of T-DXd), and at End of Treatment (i.e. at the moment of disease progression on T-DXd) patients will fill out a Swedish version of the EQ-5D-5L questionnaire on paper (15–17). The purpose of the questionnaires is to evaluate effects on HR QoL of the study-specific investigations as well as to serve as a basis for health-economic analyses of the imaging-based treatment selection approach.

#### Clinical management, outcome assessments and adverse events

Patients with HER2-low mBC in the tumor biopsy will be treated with T-DXd according to the current approvals and reimbursements. Patients with HER2 IHC 0 tumors will, irrespective of the [^68^Ga]Ga-ABY-025 PET/CT results, be treated with systemic therapy according to the choice of the treatment physician. Patients with a HER2 amplified (HER2 IHC 3+ or IHC 2+ and ISH HER2 gene amplification) tumor may be treated with T-DXd and will in that case remain in the trial and be included in the data analysis. This treatment decision is per local routines and at the discretion of the treating physician. Clinical management including decisions on supportive medication and dose reductions, is done at the discretion of the treating physician. Study subjects who have been affected by adverse events are followed until the adverse event is resolved becomes clinically insignificant, or is stabilized, or unless the subject is lost to follow-up. If a subject is lost to follow-up the Investigator must make every effort to contact the subject.

The final evaluation of the safety and tolerability of the investigational product will be performed by registering the frequency of Adverse Events (AEs), Adverse Reactions (ARs), Serious Adverse Events (SAEs), and Suspected Unexpected Serious Adverse Reactions (SUSARs). The analysis will be performed after the completion of subject enrolment.

#### Study outcomes

Objectives and endpoints are summarized in **Table 3**. The primary endpoint is the correlation between the mean [^68^Ga]Ga-ABY-025 SUVmax/peak in the five most ABY-025 avid lesions at the baseline HER2-PET and treatment response according to RECIST v1.1 after 3–4 cycles of treatment with T-DXd (Cohort 1) (12).

**Table 3:** Objectives and endpoints of the HER2-Ex PET trial.

Trial aims	Objectives	Endpoints
Primary	Investigate the therapy-predictive role of HER2-PET for the clinical benefit of treatment with T-DXd in patients with HER2-expressing mBC.	Correlation between the mean [^68^Ga]Ga-ABY-025 SUV_max/peak_ in the five most ABY-025 avid lesions at the baseline HER2-PET and treatment response according to RECIST v1.1 after 3–4 cycles of treatment with T-DXd (Cohort 1).
Secondary	Assess changes in HER2-status according to [^68^Ga]Ga-ABY-025 uptake prior to and after 3–4 cycles of treatment with T-DXd. Study the correlation between the proportion of HER2-avid metastases in relation to the total burden of disease (lesions ≥10 mm) on CT, and its prognostic and therapy-predictive value for treatment with T-DXd. Study health related quality of life at baseline, at the first response evaluation, and at disease progression. Assess the health economic impact and explore the potential cost-effectiveness of using the HER2-specific tracer [^68^Ga]Ga-ABY-025 in treatment planning in patients with mBC who are considered candidates for treatment with T-DXd	Changes (delta SUVmax/peak) in HER2-status in up to five target lesions according to [^68^Ga]Ga-ABY-025 uptake prior to and after 3–4 cycles of treatment with T-DXd (Cohort 1). Correlation between HER2-expressing total tumor volume (HER2-TTV; defined as proportion of ABY-025 avid lesions in relation to the total tumor volume (TTV, lesions ≥10 mm) defined on CT) at baseline and Overall Response Rate, Progression Free, and Overall Survival after treatment with T-DXd (Cohort 1). Change in patient reported Health-Related Quality of Life (HR-QoL) as measured by the EQ-5D-5L questionnaire from baseline to the first treatment evaluation (Cohort 1).
Exploratory	Explore differences in treatment response of T-DXd in study Cohort 1 based on PET and study-specific biopsy results (IHC 1+, 2+ and 3+; and PET SUV ≥6 vs SUV <6). Study spatial and temporal HER2-expression heterogeneity through longitudinal HER2-PET and paired tumor biopsies. Study mechanisms of resistance to T-DXd treatment by assessing HER2-status on PET after 3–4 treatment cycles (according to local clinical routines) and at the moment of disease progression (optional HER2-PET + biopsy). Study the role of HER2-PET as a potential tool for toxicity prediction (lungs, bone marrow, and heart) of T-DXd. Correlation of [^68^Ga]Ga-ABY-025 uptake and additional morphological and genomic features on tumor biopsies, such as number of HER2 gene copies.	Changes in HER2-status on PET (dichotomous [SUVmax/peak <6/≥6] as well as delta SUVmax/peak) and in biopsies (HER2-positive, HER2-low) over time and differences in these between metastases within the same patient at a specific timepoint (Cohort 1). HER2-status on PET (dichotomous [SUVmax/peak <6/≥6] as well as delta SUVmax/peak) and in biopsies (HER2-0) to assess the correlation between HER2-PET and the tumor biopsy (Cohort 1 & 2). HER2-status (SUVmax/peak <6/≥6; delta SUVmax/peak) on PET and in biopsies (HER2-positive, HER2-low at the moment of disease progression during treatment with T-DXd according to RECIST v1.1 (Cohort 1). The SUVmean, SUVpeak, and SUVmax on organs of interest (lungs, bone marrow, and myocardium) on the baseline HER2-PET and assess relation with organ toxicities according to CTCAE v5.0 (Cohort 1). The correlation between SUVmax/peak on HER2-PET to pathological findings such as number of HER2 gene copies (Cohort 1 & 2).

Secondary endpoints are related to heterogeneity in HER2 expression according to HER2-PET, changes in HR-QoL and disease-related outcomes such as PFS and OS. Exploratory endpoints relate to the relation of HER2-PET with circulating biomarkers and the possible relation with uptake on HER2-PET and the development of T-DXd-mediated toxicities such as pneumonitis.

#### Data management

Data are registered using an electronic Case Report Form (eCRF). Monitoring is performed according to Good Clinical Practice (GCP) guidelines. The eCRF provides data on patient demographics, tumor biological and treatment characteristics, as well as data on follow-up. Data are managed by the Clinical Trial Office at Centre for Clinical Cancer Studies, Karolinska University Hospital, Stockholm, Sweden. Recorded information is pseudonymized. The HER2-Ex-PET trial is registered at www.clinicaltrials.gov (NCT06830382). Ethical permission has been obtained by the Swedish Ethical Review Authority and Medical Product Agency (EU CT 2024-512721-89-00). All participants must provide their written informed consent to participate in this trial.

#### Follow-up

Follow-up will focus on the potential development of investigational medicinal product (IMP)-related effects, with medical management conducted at the discretion of the treating physician. The end of the trial is defined as the point at which the last participant in Cohort 1 has completed T-DXd treatment and has been followed for overall survival up to one year from baseline. Participants assigned to Cohort 2 will be monitored for 14 weeks following their final HER2-PET examination.

### Data analysis

#### Sample size

Based on the results from the pilot study and extrapolating from other work due to a slight difference in patient selection criteria, we expected that 70% of patients included in the HER2-Ex PET trial will have HER2-expressing tumors (IHC 1-3+) in a fresh or archived tumor biopsy and therefore be candidates for treatment with T-DXd. Of note, the patients that are potential candidates for the trial are expected to have a higher representation of HER2-low tumors than an unselected population. This figure is used solely to estimate the number of patients requiring screening in order to enroll a sufficient number of evaluable participants. Patients with HER2-low tumors (IHC 1–2+ without gene amplification) constitute the primary study population (Cohort 1) eligible for T-DXd; patients with IHC 3+ represent a small minority who may receive T-DXd at the discretion of the treating physician and, if so, will be retained in the trial and included in the primary analysis.

With an anticipated correlation coefficient of 0.45 between baseline HER2-PET signal and RECIST v1.1 response, 44 patients will be required in the primary analysis to have a power 80% and a two-sided alpha of 5%. This results in a total of 65 patients that will need to be included. To account for drop-out and technical issues, 70 patients will be included. The correlation coefficient of 0.45 is chosen as it represents a moderate correlation, which would indicate a reasonably strong and meaningful relationship between the two variables (i.e. baseline HER2-PET and RECIST response) while acknowledging the presence of unexplained variance. Sample size calculations were performed using IBM SPSS Statistics, version 30 (IBM Corp., Armonk, NY, USA), assuming a two-sided alpha of 0.05 and 80% power.

#### Statistical approach

The primary endpoint is the correlation between the mean of the [^68^Ga]Ga-ABY-025 SUVmax/peak in the five most ABY-025 avid lesions at the baseline HER2-PET and treatment response according to RECIST v1.1 after 3–4 cycles of treatment with T-DXd, and this will be assessed with Kendall’s Tau correlation coefficient. All patients are allocated to Cohort 1 and who have at least one RECIST response evaluation available are included in the primary analysis.

In order to account for HER2-expression heterogeneity and the total tumor burden of metastatic disease, a total HER2-expressing tumor volume (HER2-TTV) will be calculated as the percentage of tumor lesions >10 mm in diameter that are ABY-025 avid (SUVmax ≥6) of the total amount of tumor lesions >10 mm on CT. The Overall Response Rate (ORR) after 3–4 courses of treatment with T-DXd is then compared in patients with different patterns of HER2-expressing disease (HER2-TTV <25%, 25-75%, and >75%).

Distribution of ORR will be estimated by the Kaplan–Meier method in the HER2 TTV subgroups. For comparisons, the data will be fitted with Cox regression models, and Hazard Ratios (HRs) will be reported with 95% confidence intervals.

The patients allocated to Cohort 2, who have a HER2 IHC 0 lesion in the study-specific tumor biopsy and no documented previous HER2-low tumor samples, will not be treated with T-DXd. These patients will go off trial and no follow-up will be performed. The data from these patients, i.e. demographics and results from baseline study-specific investigations will be reported as descriptive. Explorative analyses in the correlation between HER2-PET findings and HER2-status in the tumor biopsy are planned in the whole trial population who underwent baseline study-specific investigations, as false-negative biopsies are anticipated based on earlier work by us and others (7).

Cost-effectiveness will be estimated by utilizing health economic modelling, a systematic approach to assessing the costs and effects of an intervention in comparison to the best alternative. The incremental cost and incremental effect (often expressed as quality-adjusted life years, QALYs) are used to calculate the incremental cost-effectiveness ratio (ICER). If the ICER is less than the willingness to pay for one QALY, the intervention is considered cost-effective. Analyses will be performed on observed data with no imputation.

#### Trial monitoring

Data are registered using an electronic eCRF. Monitoring is performed according to GCP guidelines. The eCRF provides data on age, body composition, tumor characteristics derived from clinical, radiological and histopathological assessment, details concerning type, dose and duration of previous and administered systemic therapy, as well as toxicity data and follow-up data on vital status. Data are managed by the Clinical Trial Office at Centre for Clinical Cancer Studies, Karolinska University Hospital, Stockholm, Sweden. Recorded information is pseudonymized. No Independent Data Monitoring Committee is included as no safety or efficacy endpoints of the IMPD are included in the trial intervention or endpoints.

## Discussion

The recognition that HER2-targeted drugs can have a clinical impact for patients who have a tumor with lower levels of HER2-expression has revolutionized how this biomarker is used in breast cancer management. Still, it is unclear with patient with HER2-low (IHC 1-2+) benefits from the HER-ADC T-DXd. PET imaging with radiotracers targeting therapeutic biomarkers offers a promising, minimally invasive approach to assess whole-body target expression in real time. The diagnostic strategy used in the HER2-Ex PET trial exemplifies a personalized treatment pathway that may improve identification of patients likely to benefit from T-DXd therapy. This could lead to enhanced treatment efficacy and reduced toxicity. Moreover, non-invasive tumor characterization supports safer and more accurate diagnostics for study participants and contributes valuable insights for future patients.

The HER2-binding Affibody molecule [^68^Ga]Ga-ABY-025, along with its analogues such as [^111^In]In-ABY-025 and closely related constructs, has previously been administered to over 200 subjects in clinical trials (EudraCT 2010-021078-12, 2012-005228-14, and 2022-500448-39-00) and published studies did not observed adverse reactions. Therefore, safety concerns are not anticipated with [^68^Ga]Ga-ABY-025 in this study. Nevertheless, all participants will be closely monitored for potential side effects of the IMP with a follow-up of at least 12 weeks after the last HER2-PET/CT investigation. Potential risks of participation include exposure to ionizing radiation, reactions to contrast agents, and bleeding associated with biopsy procedures.

At the population level, avoiding suboptimal therapies is both medically and economically essential. Treatment planning informed by predictive biomarkers helps reduce unnecessary healthcare costs and enables more efficient use of resources. Precision medicine strategies—such as theranostics, which integrate diagnostics and treatment—mark a shift toward managing cancer as a chronic condition, with the potential for long-term disease control.

Beyond the rapidly expanding indications for HER2-targeted ADCs like T-DXd, other HER2-targeted therapies are emerging, including therapeutic radioisotopes. Notably, building on the same targeting moiety as in ABY-025, a recent First-in-Human trial (NCT07081555) has begun evaluating the safety and biodistribution of [^177^Lu]Lu-ABY-271 in patients with HER2-positive mBC, representing a promising new avenue in HER2-directed treatment.

Conducting a multicenter PET trial with a novel radiotracer like [^68^Ga]Ga-ABY-025 necessitates a harmonized, multidisciplinary effort at each site. This includes aligning expertise, coordinating trial logistics, standardizing investigational medicinal product preparation, and ensuring data consistency across centers. Establishing best practices and demonstrating clinical utility, cost-effectiveness, and workflow feasibility are essential for broader implementation as a diagnostic selection tool in routine care.

Several confounding factors may influence interpretation of the trial findings. Variability in prior treatment history, tumor burden, and metastatic pattern may independently affect T-DXd response irrespective of HER2-PET signal. Despite a harmonized acquisition protocol, inter-site differences in scanner hardware and courier-delivered tracer to one site may introduce systematic variation in image quality and tracer specific activity. The variable interval between baseline and follow-up HER2-PET — depending on whether patients complete three or four T-DXd cycles before reassessment — may affect comparability of dynamic PET findings. Concurrent corticosteroids may further modulate receptor expression and tracer uptake independent of true HER2 status. Finally, anticipated expansion of T-DXd indications to earlier treatment lines and HER2-ultralow tumors during the conduct of the trial may introduce additional heterogeneity in patient characteristics and treatment context that could complicate interpretation of results.

In conclusion, the HER2-Ex PET trial is, to our knowledge, the first clinical study to evaluate the predictive value of HER2-targeted PET imaging with [^68^Ga]Ga-ABY-025 in patients with HER2-low mBC receiving T-DXd. The integration of biopsy-based assessment, health-economic analysis, and translational research will further elucidate the clinical and scientific value of molecular imaging in modern oncology.
